# Outsiders, insiders, and intermediaries: village health teams’ negotiation of roles to provide high quality sexual, reproductive and HIV care in Nakaseke, Uganda

**DOI:** 10.1186/s12913-019-4395-4

**Published:** 2019-08-13

**Authors:** Samantha Perry, Cynthia D. Fair, Sahai Burrowes, Sarah Jane Holcombe, Robert Kalyesubula

**Affiliations:** 1 School of Social Service Administration, University of Chicago, Chicago, IL USA; 2 Professor and Chair of Public Health Studies, Elon University, Elon, NC USA; 3 Associate Professor, Public Health Program, Touro University California, Vallejo, CA USA; 4 University of California, Berkeley, CA USA; 5 Makerere University College of Health Sciences, Kampala, Uganda; 6 African Community Center for Social Sustainability (ACCESS), Nakaseke, Uganda

**Keywords:** Village health teams, Uganda, Roles, Sexual and reproductive health, HIV care

## Abstract

**Background:**

Community health workers, known as Village Health Teams (VHTs) in Uganda, play a central role in increasing access to community-based health services. The objective of this research is to explore tensions that may emerge as VHTs navigate multiple roles as community members and care providers particularly when providing sensitive reproductive health and HIV care.

**Methods:**

Twenty-five VHTs from a rural clinic in Uganda completed semi-structured interviews focused on experiences providing services. Interview questions focused on challenges VHTs face providing services and strategies for improving quality care. After translation from Luganda and transcription, interviews were analyzed using content analysis to identify emergent themes.

**Results:**

Most VHTs were female (*n* = 16). The average age was 46, and average length of VHT work, 11 years. Analyses revealed that all VHTs capitalized upon the duality of their position, shifting roles depending upon context. Three themes emerged around VHTs’ perceptions of their roles: community insiders, professional outsiders, and intermediaries. A caregiver “insider” role facilitated rapport and discussion of sensitive issues. As community members, VHTs leveraged existing community structures to educate clients in familiar settings such as “drinking places”. However, this role posed challenges as some VHTs felt compelled to share their own resources including food and transport money. Occupying a professional outsider role offered VHTs respect. Their specialized knowledge gave them authority to counsel others on effective forms of family planning. However, some VHTs faced opposition, suspicions about their motives, and violence in this role. In balancing these two roles, the VHTs adopted a third as intermediaries, connecting the community to services in the formalized health care system. Participants suggested that additional training, ongoing supervision, and the opportunity to collaborate with other VHTs would help them better navigate their different roles and, ultimately, improve the quality of service.

**Conclusions:**

As countries scale up family planning and HIV services using VHTs, supportive supervision and ethical dilemma training are recommended so VHTs are prepared for the challenges of assuming multiple roles within communities.

**Electronic supplementary material:**

The online version of this article (10.1186/s12913-019-4395-4) contains supplementary material, which is available to authorized users.

## Background

Sexual and reproductive health (SRH) concerns, including HIV, represent some of the most pressing health issues in Uganda and sub-Saharan Africa as a whole. Although the country is widely considered an HIV success story for its vigorous response to the epidemic and the resulting reduction in rates of new HIV infections, HIV remains a significant health burden, with approximately 1.5 million adults and children in the country infected [1]. Uganda has one of the most mature antiretroviral drug treatment programs on the continent, but significant gaps in treatment coverage remain. As of 2016, approximately 33% of adults and 53% of children living with HIV were still not receiving treatment [1]. Both HIV prevention and treatment programs in Uganda have been characterized by the heavy involvement of community-based organizations and community volunteers in order to extend services to the country’s predominantly rural population. Limited awareness about personal or partner HIV status, and a lack of effective and acceptable female controlled method of prevention are two of the main challenges faced in the implementation of the Ugandan National HIV and AIDS Strategic Plan [2].

Uganda also has one of the world’s highest fertility rates (5.01 children per woman) with 35% of Ugandan women reporting an unmet need for family planning and 1.2 million women having unintended pregnancies each year [3]. Lack of access to sexual and reproductive health services is particularly acute for adolescents whose unmet need for family planning is estimated to be 60% [4].

Most of the efforts to scale up SRH services and other services in Uganda have focused on the supply side at the health facility level – training workers and increasing supplies [5]. However, in an effort to improve linkages between facilities and communities, the Ugandan Ministry of Health introduced a “task sharing” strategy that uses community health workers (CHWs) organized into Village Health Teams (VHTs) to bring services closer to the community and increase the sharing of health information. VHTs or CHWs were introduced in 2001 to help address maternal and child mortality challenges. VHTs are drawn from the communities that they serve and, with training, deliver a variety of health education and primary health services. Although a range of compensation arrangements for CHWs exist, in most countries CHWs are often unpaid volunteers. This is frequently the case in Uganda, there are initiatives to provide modest compensation for VHTs [6]. VHTs in Uganda are equivalent of “Health Centre 1” in a five-tiered health center system and are responsible for the health of community members at the household level [7]. They serve as a community’s initial point of contact for health and social services, help to build community members’ social capital and understanding of basic health services, and facilitate their access [8].

The use of CHWs to deliver health education and health care has a long history but has gained popularity recently as part of a larger movement throughout low- and middle-income countries to “shift” or share health care delivery tasks from scarce, highly-skilled health workers such as physicians to mid-level health workers and community members. [9, 10] CHW initiatives such as Uganda’s VHTs have proven to be an important health care delivery strategy, particularly for underserved rural populations. However, tensions around rights and roles may emerge, as VHTs must navigate their roles as both community members and health care deliverers [11]. This study explores the benefits and challenges VHTs experience as they utilize multiple roles to provide high-quality sexual and reproductive health care.

There are divergent views about task sharing in health care to the community level. Some have argued that it is a mechanism of mobilizing community resources to most effectively reach clients [12]. Others fear that community health workers will just be viewed as another pair of boots on the ground, and will be not be utilized to their full potential since they often lack formal recognition, and are undervalued, under resourced, and undercompensated [13]. In addition, scholars have delineated concerns about how these workers interact with the community [13–15]. Empowering and mobilizing community members to deliver care was designed to protect the rights of clients and respect the autonomy of communities, as well as to expand their access to services. However, utilizing volunteers as health care deliverers brings a new set of challenges around client rights. VHTs must negotiate the duality of their position as an insider in the community and a deliverer of messaging that may contradict their communities’ values. Social and cultural norms, values, practices, and beliefs are all contextual factors that affect VHT performance [16]. Further, tensions exist concerning the payment of VHTs related to the financial sustainability of programs, as well as the worry that formalizing the “helper” role will undermine community trust [11, 17].

Role identity theory describes the ongoing socially constructed process, through which individuals give meaning to themselves within their particular roles [18]. Roles are seen as fluid sets of relationships, the social feedback VHTs receive within their role shapes the identity they construct, as well as their future behavior [19]. How other individuals respond to the person occupying the role will influence how they perceive their role and their own identity. VHTs are in a particularly unique position as being both representatives of their community and the health system. Since their position is by nature, more ambiguous and not as defined as that of a health professional, VHTs are involved in a continuous renegotiation of their roles and who they perceive themselves to be [18].

VHTs take on a number of quasi-formal roles as part of the health system including patient support activities (counseling, home-based care, education, and adherence support), as well as health service support (screenings, referral to services) [20]. However, studies show that they are usually involved in informal activities as well such as assisting with meal preparation, bathing, performing household chores, and listening to neighbors’ problems. [21–23]

There is a risk that if not managed, the adoption of a VHT role and the quasi-professional status it offers may generate an identity of superiority, distancing the VHT from her or his community, and may lead to unintended negative consequences for the implementation of VHT programs. Although they are not technically government officials, CHWs such as VHTs, have been described as “street level bureaucrats”, providers who can use their autonomy to reinterpret program policies according to their personal priorities [24]. This provider behavior is a form of “clientelism”, providing clients with individualized access to resources/knowledge in exchange for their support for the VHT [25].

Issues around family planning, sexual and reproductive health, and HIV can be particularly controversial in communities. In a western Kenyan community, where contraceptives were thought by many community members to cause sickness, death, or infertility, CHWs modified their advice to deviate from their training in order to not contradict the local values or be blamed for illnesses [25]. VHTs’ concerns for a healthy community through family planning and HIV care, as well as their concern for respect for themselves, may be in conflict with one another, and this tension could affect the care they deliver. These conflicts may be particularly acute with regard to reproductive health and HIV counseling because of ongoing stigma surrounding HIV, and the confluence of beliefs around pregnancy, childbirth, infant care, and sexual behavior that must be addressed in counseling women and their families [26].

It is necessary to understand VHTs’ perceptions of their roles within their community and the tensions that may arise, as these contextual factors may influence the quality of care provided, retention rates, and overall VHT satisfaction. Previous research in Uganda has explored clients’ perceptions of reproductive health services and adherence to HIV protocols, as well as perceived barriers to accessing services. [27–32] Relatively few studies have examined how VHTs perceive their roles, and how their health work intersects with their other identities as neighbors, friends, and community members. Recognizing how and why these roles are adopted and practiced is critical in promoting VHT recruitment and retention, intervention efficacy, and program success. Using role identity theory as a framework, the objective of this study is to examine how VHTs negotiate and perceive their roles, particularly when providing sensitive reproductive health and HIV services. Specifically, we seek to understand what tensions emerge for VHTs as they navigate their different roles as community members and health volunteers, and how they manage these challenges to provide quality care.

## Methods

### Participants

This qualitative study was conducted at the African Community Center for Social Sustainability (ACCESS) in the Nakaseke District in central Uganda. ACCESS is a community-based organization dedicated to working with vulnerable populations to alleviate poverty and illness. Participants were sampled purposively in order to obtain responses from as many villages and rural areas as possible. VHT members over the age of 18 who had been working for at least one year were invited to participate in a one-time interview. ACCESS staff recruited eligible VHTs and a total of 25 participated. All those who were approached about the study agreed to participate.

### Procedure

Recruitment occurred over 2-weeks and interviews took place at ACCESS during a 6-week period in May–July 2017. A semi-structured qualitative interview guide was used to explore the VHT’s views on patients’ rights with regards to sexual and reproductive health (SRH) services and HIV counseling, and to identify the challenges they face and the strategies they employ to overcome those challenges when delivering services. Additionally, the interview guide elicited suggestions for how the VHT program could be improved. Specific interview guide questions focused on the perceived role and responsibilities of the VHT, their handling of potentially difficult cases regarding HIV and SRH services, and their approach to work within the community setting. Please see Additional file 1 for interview guide. The questionnaire was designed in English, translated, and then pre-tested before the actual data collection by administering it to a total of 5 VHT members. Participants were interviewed individually. Interviews were conducted in the local language (Luganda), by the primary investigator (SP) and a translator in a private location within the health care clinic. Interviews typically lasted one hour.

Respondents were reimbursed 10,000 Ugandan shillings (approximately US$3.00) to compensate for their time and travel. Before interviews, participants were informed of the purpose, risks, and benefits of participation, and written informed consent was obtained. Signed consent forms were stored in a secure location. Institutional review boards at Elon University (# 00002430), Touro University (# PH-7717-TW), and the Mulago Hospital Research and Ethics Committee (#1142) approved the study.

### Analysis

Interviews were audiotaped with permission from the participant and transcribed and translated. Transcripts were reviewed for accuracy and entered into Dedoose, qualitative data analysis software. Data analysis was conducted using an iterative coding process [33]. The data was coded preliminarily by the primary investigator (SP) during the period in which interviews were being conducted. The interview guides were revised as a result of initial interviews in order to follow up on emerging themes. After data collection was completed, SP and CF began with open coding, and moved to more refined codes, subthemes, and then themes. Interviews were thematically analyzed and coded using a constant comparison method [34]. The codes were applied to interview transcripts and summary statements with representative quotes were developed for each theme. These codes were confirmed with the other investigators through conference calls and emails. Analyses took place from September 2017–January 2018.

## Results

The majority of VHTs were female 64% (*n* = 16), with an average age of 46 years, and of 11 years working as VHTs. All of the participants had received some formal education with over half (*n* = 15) reaching some level of secondary schooling. Forty percent of VHTs interviewed (*n* = 10) participated in a family planning program in which they had received training in family planning, and were eligible for incentives based on their number of clients electing to use family planning services. See Table 1 for additional demographic features.

**Table 1:** Participant Demographics

Demographic Variable *n* = 25	
Age	Mean = 45.5
Gender	
Male	9
Female	16
Marital status	
Married	21
Unmarried	2
Widowed	1
Divorced	1
Has Children	25
Education	
Some primary	3
Completed primary	7
Some secondary	13
Completed secondary	2
Participant in ACCESS Family Planning Program	10

Three themes emerged around VHTs’ perceptions and construction of their roles: VHTs as community insiders, professional outsiders, and as intermediaries. All VHTs capitalized upon the multiplicity of their positions, shifting roles depending upon the particular challenges they faced when providing services. When tensions emerged due a perceived identity or characteristic of a particular role, VHTs would adjust their behavior and activities in order to mitigate the strain. Each role offered benefits in working with clients around culturally sensitive health topics such as SRH and HIV, but also posed particular challenges. Table 2 provides a concise overview of each role, related characteristics, as well as examples of how the roles manifested in daily work. Finally, participants expressed the desire for additional support to address challenges of their role negotiations including continued training, regular supervision, and more formal collaboration with other VHTs.

**Table 2:** Themes: Roles of Village Health Team Members

Role	Characteristics of Role	Manifestations of Role
Insider	Caregiver/friend	Rapport-building with clients and community; development of clients’ sense of social connection. Altruistic motivation
	Work within community structures	Education of clients in churches, drinking places, pool halls; enhancement of clients’ family support systems; provision of ongoing and consistent follow-up care; reduction of community-level stigma
	Client and community member mistrust	Addressing clients’ concealment of HIV status by lying about own HIV status to increase relatability
Outsider	Professional	Possession of specialized training and knowledge; fulfillment of responsibilities including client education, counseling on medicine and family planning methods, participation in door-to-door community mobilization, aid with antenatal care, instruction on community sanitation, work to ensure client HIV medication adherence; collaboration with other VHTs and medical workers. Increased VHT self-esteem as a result of work
	Representatives of government	Feeling a sense of helplessness when faced with community members’ frustration about the lack of public resources
Intermediary	Advocate	Notifying hospital of disease outbreaks; working to ensure accountable hospital care; advocacy for client needs
	Bridge	Simplification of medical terms; assistance to clients in navigating health care system; use of hospital visits as way to bring clients for HIV testing; education of other community members
	Gatekeeper	Referring clients for services; arbitration of which clients are referred
	Not fully a health professional	Lack of personal respect from health facility medical workers: medical workers’ discarding of referrals forms and refusal to serve patients referred by VHTs. Resultant diminishment of client trust in VHT work and role

### Theme 1: VHTs as insiders

As a community insider, VHTs were seen as a caregiver or friend and able to adeptly work within existing community structures to relay health information and provide services. However, this insider position sometimes led to community member mistrust, as clients feared that VHTs might violate patient confidentiality.

#### Subtheme: caregiver/friend

The primary impetus for most of the VHTs to become volunteers was their desire to promote the well-being of their community. They were intrinsically motivated by a sense of altruism, volunteering their time and effort “for the good of (their) fellow citizens.” All participants indicated that they felt positively about being chosen to be a VHT, and most proactively sought out their roles.

This sense of benevolence was also a hallmark feature of their being a VHT. As a neighbor and fellow community member, participants described going far beyond their professional role as a health educator. Over half of the participants mentioned using their own resources to help clients by fetching water, cooking food for those too ill to move, accompanying sick patients to the hospital, and providing transportation for those who could not afford the cost:

*“Like when I have reached their home and see she is sleeping. I will go closer to her and ask what the matter, if she can’t sit I give a hand and if she wants food to eat but is not capable of cooking then I will ask for permission to go and bring food from my place for us to eat together.”* (Participant #21, female, Non-ACCESS VHT)

*“I make sure they eat well, give them comfort. I used to do it every morning for this specific person, cooking, but he died a few weeks ago. The woman (his wife) was drunk and wasn’t cooking for him. The woman is in bad shape, I told her to go for testing but she won’t go. I feel bad because they have responsibilities in the community, they are good people.”* (Participant #18, female, Non-ACCESS VHT)

As an insider in their communities, VHTs utilized their unique position to help build rapport with their neighbors and new clients. Participants described easing potential clients into discussions about sensitive topics, such as sexual and reproductive health and HIV, by first establishing a *“friendship”* (Participant #1, female, ACCESS VHT). Kindness, listening, and discussing ordinary matters were key features of their initial conversations with clients. One participant stated, *“I make sure that I have a strong relationship with them so that they are able to express themselves to me,”* (Participant #20, male, ACCESS VHT) and another noted: “*also some of the community doesn’t know me, but when I talk to those people, I get to be known. I try to get them to start trusting me, I get to know their kids*” (Participant #12, male, ACCESS VHT). By first building trust with community members over every day communal matters, VHTs were able to establish the foundations of a working relationship in which they could bring up more culturally sensitive health information.

Many participants pointed out that the challenges they faced were similar to that of their patients, such as problems with farming, a lack of money, and having to care for many children. The VHTs’ ability to identify as ordinary community members created a sense of social connection with their clients. For example, when counselling HIV-positive patients, they were able to hold themselves up as an attainable example of someone who is still living and even thriving while being HIV-positive. One participant stated,

“*I will take an example of me, that look at me I am taking my medicine, doing the community work, taking good care of my family, paying school fees, cultivating food for the whole family, and even getting some money, so if you take your medicine well you will be able to have a good future for your family.”* (Participant #4, female, ACCESS VHT)

#### Subtheme: working within community structures

An indispensable feature of VHTs insider role was their ability to work within existing community structures to tailor their SRH education to their audience. Many VHTs discussed going to communal spaces within the district to do initial education outreach. They held both formal and informal trainings at pool tables, after church, and in schools with youth. These communal spaces were mentioned specifically around the topic of family planning, which many VHTs stated men did not allow their wives to use because of cultural beliefs about the prestige of having many children and myths surrounding the side effects of family planning methods. One VHT explained,

*“I always go and meet with men at their hangouts, like drinking places. I talk to them about it casually in the group. Those whose interest is piqued will find me somewhere when it’s just the two of us. They say ‘you know what you were talking about earlier, the family planning, I am interested, you tell me more.’”* (Participant #12, male, ACCESS VHT)

While health professionals would not necessarily know, or be able, to utilize these spaces for educational outreach, the VHTs’ insider role gave them both the knowledge and the means to effectively discuss family planning and SRH with women as well as men, who often hold much of the power in the family unit, and may influence whether or not their wives access such services.

VHTs also used their position to work with patients’ entire families regarding preventing and treating HIV. Many VHTs discussed how being a known member of the community afforded them the ability to act as a counselor on such issues. They often worked with the HIV-positive partner to inform the other, *“we join together and teach him what is going on … we keep on counseling him slowly by slowly”* (Participant #1, female, ACCESS VHT). In cases where one family member was too scared to tell their partner, the VHT was often able to act as a neutral third party, and discreetly work to get the other partner to go for testing without disclosing the status of their client, *“I deal with the [HIV] negative partner so that he/she goes for a blood test”* (Participant #20, male, ACCESS VHT).

Participants described utilizing the family as a support system in caring for HIV-positive clients. For example, to ensure medication adherence, participants discussed training a family member such as a wife, husband, child, to assist in reminding their HIV-positive clients to take their ARV tablets.

*“The victim needs to be near any family member, who can always tell him/her to take medicine in time, prepare him meals and always be on his neck so that he takes medicine appropriately. This village is so big I might not be in the position to avail myself in time. So I advise the family member who can take good care of him/her*” (Participant #6, female, Non-ACCESS VHT)

*“First, what we always do is because you might not be in your home, what you have to do is to get someone like in his family or chose someone in a private way and ask them to take care of that person to take his medicine, teach them why they have to take the medicine, what will happen if they don’t. You say ‘so let’s join hands and look after him and tell me if he’s taking the medicine or not, if not then more counseling is to be done. Show love by not shouting at them to please take your medicine. No, show love and provide them with what to eat … love is the most important thing.”* (Participant #22, male, Non- ACCESS VHT)

Apart from working with the family to care for HIV patients, VHTs also worked at the larger community level to break down stigma and make life better for their clients. Since the VHTs have a deep knowledge of the cultural beliefs of their communities and the sources of stigma, they can target their education to those who exhibit such behavior. One participant stated,

*“I make sure that she or he gets the best comfort ever, as a VHT, we normally do not allow discrimination among our people. Stigma is common in the community. You find that the victim has that fear of how the community would react when found out about their sickness, and in most cases this prevents them from associating with other people. It’s our responsibility to counsel such people and try to make the community see that HIV is not a killer disease especially when given the best care, treatment, love. In order to increase on their life span, they need the communities’ support.”* (Participant #5, female, ACCESS VHT)

One participant who had been working for twenty years with HIV patients in her community stated that because of her targeted work, *“they no longer fear, no longer hide … now they live with no worry. They no longer get tortured by people that so and so is sick.”* (Participant #21, female, Non-ACCESS VHT).

Being a neighbor and working within their own community gave VHTs the ability to follow-up consistently with clients. When clients refused or were apathetic to the HIV counseling, VHTs simply persisted in their role, consistently going back and discussing the topic with clients, *“I first become close and create that friendship … I don’t leave the matter on that day for her to decide. No, I take up my role and go back to repeat the same issue we had talked about, and then I send her to the hospital”* (Participant #3, female, ACCESS VHT). Part of their willingness to follow-up so ardently with clients stemmed from their recognition of where they started out in their own knowledge of such topics, and of how it takes time to break down misconceptions around SRH topics. When individuals refused to listen, VHTs discussed how they stayed calm and came back to the topic later,

*“Even in family planning, we have those who oppose us, but we stay cool. You go ahead now there are even men who do support, they approach me and tell me that ‘ahhhh what you did helped me a lot now my child has grown. even in income we no longer suffer, I don’t know whether my wife told you, but I want her to come back.’ It doesn’t become a war if a person has refused because may be he/she doesn’t know, even we didn’t know in the beginning we were just trained.”* (Participant #3*,* female, ACCESS VHT)

#### Subtheme: client and community member mistrust

Despite VHTs coming from the community and believing themselves to be caring and trustworthy, a theme of mistrust emerged from the data. It is clear that there is tension between VHTs being community members and also members of the health system. Participants discussed how often people from their community would go to the hospital to be tested, but would then hide their status from the VHT in the village, *“they fear other people knowing (their HIV status)…when they go back to the community, they won’t open up”* (Participant #18, female, Non-ACCESS VHT)*.* These individuals were concerned that the VHT would violate patient confidentiality.

To combat this lack of trust, some VHTs tried to increase their relatability and decrease the patients’ perceived sense of stigma by lying about their own HIV status. One participant said,

*“If she says no, then I will tell her that we are all sick that I am also sick. I put myself in her situation that it is not the end, so if you go and test for HIV … All people are sick and if you test you always know the truth … if you don’t test when you have it you will have a terrible death. What we want you to know is that all people are sick and medication is there.”* (Participant #1, female, ACCESS VHT)

Another participant discussed how he lied about his status to break down stigma between family members who were arguing about who was infected first, *“It happened in three families I lied to them and said I am also HIV-positive, I don’t know if me or my wife brought it, but what you’re supposed to do is focus on your children and taking care of them.”* (Participant #20, male, ACCESS VHT).

### Theme #2: VHTs as outsiders

VHTs also had to work within formal spheres with other professionals, and as such were sometimes perceived by others and themselves as being outsiders. A characteristic of this outsider role included the perception of VHTs as professionals, which conferred status and access to social capital resources. However, a negative characteristic of the outsider role was being viewed as a representative of the government.

#### Subtheme: professional

VHTs by definition are part of Uganda’s health care system; they are ‘Health Center I’ and represent the government in their roles. They are recognized as having specialized knowledge and training. One participant summarized their role saying, *“I have to be exemplary and I have to attend all health seminars so that I pass the information over to the community. Musawo means medical person, I need to act as a good VHT”* (Participant #20, male, ACCESS VHT). As part of their formal work, VHTs educate clients, counsel them on medicine and family planning methods, participate in door-to-door community mobilization, assist in antenatal care, teach people about sanitation (latrines, drying racks, rubbish pits), ensure HIV medication adherence, and desensitize the community to topics such as HIV and SRH.

Their perceived professional status gave them access to social capital resources, many VHTs talked about collaborating with other VHTs or formal medical professionals to assist in their SRH work. When clients refused HIV testing or were resistant to hearing about family planning, one participant said, *“I will invite another VHT and combine ideas. I pay a visit to the (client) and try to make them understand. I work with other VHTs when I need help”* (Participant #13, female, Non-ACCESS VHT). Some VHTs also worked directly with health care clinics as part of their duties. They assisted with patient intake, filing paperwork, and buying and distributing medicine.

The outsider role of VHTs gave them an increased sense of self-esteem and empowerment. They felt distinguished amongst their fellow community members, *“whenever I pass in the community they always call me musawo, or medical person. I like to be called that it’s an honor*” (Participant #12, male, ACCESS VHT). Participants also mentioned that after they became a VHT people treated them differently, that it “uplifts” them.

#### Subtheme: representatives of the government

As representatives of the government, VHTs were often met with anger and frustration. When one VHT informant was conducting malaria education in their community, a client said “*… ‘instead of the government wasting time giving out stupid nets they should at least do something better.’ As a VHT I advised them to use (the net) because they will get sick and die, and that stupid government will not even know about them*” (Participant #1, female, ACCESS VHT). Additionally, community members often became angry and even violent with VHTs when there was a lack of resources, “*When people ask for something from me but I don’t have the power or the money to give it to them … they get very angry with me, and I try to comfort them. I have to wait on organizations and the government”* (Participant #9, female, Non-ACCESS VHT). Several participants mentioned being chased or physically harmed by angry community members. Some people also believed that the VHTs were stealing government provisions from the community, “*the community thinks that we are being paid and just eat the money given to them*” (Participant #21, female, Non-ACCESS VHT). VHTs felt a sense of helplessness in these situations and expressed feeling “pressure” if they were not able to meet the monetary and resource demands of their clients.

### Theme #3: VHTs as intermediaries

In trying to balance both an insider and an outsider role, VHTs assumed a third position, that of intermediary, which could take the shape of serving as advocate, a bridge or a gatekeeper. They attributed the improvements in community health to their role, *“Long ago people used to not go to the hospital when they fall sick, but nowadays things have changed and this is due to the trainings we get to share the information to the community”* (Participant #5, female, ACCESS VHT). However, the challenge of this intermediary role is that VHTs were perceived to be not fully health providers by professional medical workers, leading to tension and a lack of respect.

#### Subtheme: advocate

The VHTs’ unique position as a trained health worker with an understanding of community life allowed them to advocate for their clients’ needs. Eleven participants mentioned advocacy as part of their responsibilities to their patients. One participant described themselves as a *“chain between the hospital and the community … I do advocacy knowing the communities’ problems and taking the problems to responsible people to find solutions”* (Participant #18, female, Non-ACCESS VHT). When there were outbreaks of diseases like TB, the VHT would notify the hospital and transmit health information to the community. When patients reported that the care they received at the hospital was inadequate, the VHTs would go up the chain of command and report to the hospital superiors.

#### Subtheme: bridge

VHTs capitalized on their dual roles as insiders and outsiders to operate as a bridge between community members and the formal health care system. When community members come to them with an ailment, the VHT would help the patient decide what level of care they need – a simple first aid fix, a health center visit, or a trip to the regional hospital. They would often simplify medical jargon or prescriptions for clients, as well as continually remind illiterate patients about what their drug prescriptions were. Most VHTs described accompanying their clients to the hospital in order to ease their fear or assist them in navigating the often overwhelming formal health care systems, *“If the person is scared of going, I escort them to the hospital … When I take him/her to the hospital I talk to the medical worker to counsel that patient”* (Participant #13, female, Non-ACCESS VHT). When VHTs felt that their knowledge was not sufficient to counsel the patient to take their antiretroviral medications or test for HIV, they would bring that individual to the hospital guiding “*… him/her to the professional counselors for extensive counseling”* (Participant #6, female, Non-ACCESS VHT). One VHT who worked with antenatal care noted that, “*When I see a pregnant woman, I make sure I go to the hospital with that woman to make sure she goes. If found HIV-positive she will take medication, and I make sure she gets the right kind*” (Participant #13, female, Non-ACCESS VHT). VHTs also used their access to the hospital as a way to get people tested for HIV who were not otherwise inclined. They would refer them for their other illnesses, accompany them to the hospital, and then with the assistance of the medical worker, counsel them on the benefits of knowing their status. One VHT mentioned extending this role as a bridge between the hospital and the community by involving more community members in health education.

*“Now we have this process going on to extend medicine to the people in the community, we tell them to reach six in number for us to bring the medicine near to them to ease on the transport issue. By that we are trying to create a good relationship between the community members and the VHTs”* (Participant #4, female, ACCESS VHT).

#### Subtheme: gatekeeper

As a third party, VHTs also acted as gatekeepers to services and held some power in that regard. They could decide who to refer to services, and who not to refer. For example there is a local Orphan and Vulnerable Children (OVC) program that provides food, income supplements, and job training for children who are refereed. VHTs who work within that town decide which children to refer for help. In some cases, *“they (the community) are so proud that they chose the right person who hunts down good things for them”* (Participant # 7, male, Non-ACCESS VHT).

#### Subtheme: not fully a health care provider

A challenge of the VHTs’ intermediary roles is that they were never viewed as full-fledged health care providers. Several participants described facing a lack of respect from medical workers, and said that this was one of the greatest challenges in preventing and treating HIV. One participant said,

*“Sometimes when we refer people to the hospital, they are not attended to by the medical workers. This affects our work as VHTs. I might refer someone to the hospital, but when she gets there the medical workers they won’t help. They don’t keep track of the referral sheets and just toss them aside, they don’t give them medication. So when I refer someone, they come back and say ‘They didn’t help me.’ And then they won’t go back. Some medical workers won’t help the patients I refer. They will just say, ‘For us we are already tired.’ They have a different attitude towards VHTs. I feel minimized by medical workers. They think us VHTs are stealing their duties, that we are competition.”* (Participant #13, female, Non-ACCESS VHT)

This caused patients to become frustrated, “lose trust”, and view the VHT as “incompetent.”

### Theme #4: additional support

When asked what would improve their work in meeting the challenges of role negotiations, VHTs requested training, supervision, and formal methods of collaboration between fellow workers. Non-ACCESS VHTs cited ACCESS’ family planning program as an example of training they wanted to participate in,

*“All we need as VHTs is motivation in terms of meetings. ACCESS trains VHTs but they only take some of us. For example, they want twenty VHTs to do family planning training, but I didn’t get chosen. I need more help, and training. Meetings to learn and to talk with other VHTs.”* (Participant #10, female, Non-ACCESS VHT)

While this was a request of VHTs who were not a part of ACCESS’ program, even those who were ACCESS VHTs stated a need for more regular trainings and meetings. One participant said,

*“We need more training to improve on our skills, as well as boosting our relationship with the organizations. The relationship between VHT and organizations like ACCESS should be improved. There is no regular training for VHTs, no one to encourage us. We need a space to talk with other VHTs. If there were more training I would be more active. If you are just at home, you forget responsibilities; you forget what you’re supposed to be doing, it’s easier to not try to manage responsibilities for both your work and your home.* (Participant #11, female, ACCESS VHT)

## Discussion

This study provides insight into how VHTs view their position within the community and the health system, and their perspectives on how the community and health system view them. As VHTs move through their work they occupy both insider, outsider, and intermediary roles depending on the context, and their relationship to the client and health system. In providing SRH and HIV care, they must navigate these various roles, which confer both access and benefits, as well as barriers and challenges to their work. Findings confirm previous research that VHTs assume a variety of roles including patient support both formal and informal [15, 16]. However, there was no evidence of “clientelism” where VHTs provided services in exchange for other goods or benefits as found in earlier research [20]. Although the latter finding is heartening, we must note that it might result from only having the VHTs’ perspectives. Had we studied community perspectives of the roles and behaviors of VHTs, we might have found greater evidence of clientelism.

VHTs constantly renegotiated their roles to fit the challenges of their context - a low resource, rural health care system with cultural stigma around HIV and SRH. Role identity theory holds that within a role, an individual will respond to relational feedback and their environment, to behave or make decisions that best fit the context within which they are operating [13]. Sometimes this response includes a shift from one role identity to another in order to best address the challenges present. In their position as VHTs, participants had to constantly shift from role to another, re-negotiating who they presented themselves to be and from which role they operated. These role identities of a community insider and professional outsider were sometimes contradictory. As a result, VHTs adopted a third role as an intermediary.

The insider role was characterized by the VHT being seen as a caregiver and a friend. Altruism, and a concern for the health of their community was the impetus for most VHTs to join the health system. This can be seen as an extension of the people’s spirit of ‘ubuntu’ or kindness, a prevalent social norm in rural African communities [13]. The insider knowledge they had about their community allowed them to work within existing structures such as churches and social gatherings, as well as partner with families to support HIV-positive individuals. In line with previous research, much of the VHTs work was outside of their traditional position description, and included assisting sick clients with food, bathing, and monetary needs [23]. A new finding here not previously explored in the literature, is VHTs’ responses to a perceived sense of community member mistrust. When individuals hid their status because of a fear that confidentiality would not be kept, VHTs attempted to increase their relatability by pretending to also be HIV-positive.

VHTs were not able to always identify with the people they were serving. In keeping with the design of their role as part of Uganda’s task shifting, VHTs often identified as outsiders, professionals within their community. They were able to perform tasks traditionally associated with educated health workers including antenatal care, community mobilization, family planning education and services, HIV counseling and assurance of medication adherence. Previous research suggests that VHTs are able to do these professional tasks effectively because “the community members are able to accept their teaching, and they become flexible and willing to be taught” (p. 499) [22]. A challenge within this outsider role was that VHTs were viewed as representatives of the government, and community members and, at times, directed their anger at the government towards VHTs.

The VHTs often adopted a third role as intermediaries, acting as advocates within the health system, bridges between communities and services, and gatekeepers to services. Some of the tasks VHTs described within this role have been noted in previous research, including translating from local languages, accompanying clients to the hospital, and improving communication between patients and providers. [35–37] Where this role is supported, VHTs can become significant players in ensuring patients receive high-quality HIV and SRH care [38]. However, this study found that VHTs playing this middle role faced disrespect from medical workers, with some saying that this was the greatest challenge they faced in their HIV and SRH work. Previous research has described the hierarchical relationship between health professionals, such as between nurses and VHTs, within the hospital setting. Nurses have worked to mark their territory as professionals, resisting recognition of VHTs in the form of provision of uniforms or inclusion in hospital staff meetings [18]. In this study, VHTs reported medical professionals threw out VHT patient referral forms and refused to see patients that VHTs had escorted to the health facility for services. As a result, patients became frustrated with VHTs and lost confidence in their ability to work on their behalf.

In facing the challenges of navigating their many roles, VHTs requested regular training, supervision, and meetings with other VHTs. Previous research has argued that failures of VHTs to link community members to health care is due to issues in the power relations among health care system stakeholders and a lack of partnership between VHTs, medical workers, and health organizations [8]. As VHTs operate in often ambiguous spaces, moving between outsider, insider, and intermediary positions, proper supervision, meetings with stakeholders, and greater local ownership of the VHT program is necessary to counteract the barriers VHTs face in motivation, retention, and providing quality care.

### Methodological considerations

This study was conducted at a single organization thus limiting generalizability to other VHTs in Uganda. However, themes resonated with previous research. Social desirability bias is possible, as participants may have exaggerated their responses to present themselves in the best light. The use of an interpreter also may have influenced what the participants shared during the interview and nuances of the local language may have been lost due to translation.

## Conclusion

Despite some limitations, this research sheds light on how VHTs construct their identities, constantly renegotiating their roles to best address the challenges faced within their ambiguous position. As both health care providers and community members delivering care and education that is often culturally sensitive, they adapted to fit the needs of clients – shifting between an insider, an outsider, and an intermediary. The VHT model is a low cost way to scale up sexual and reproductive health care for rural communities, however as programs are implemented the health system must be mindful of role tensions that may arise. Future research should consider how community members view the care they receive from VHT members, as well as how health care professionals view their relationship with VHTs. As countries work to scale up rural health services through VHTs, three related recommendations emerge from the research here. VHTs’ roles and contributions can be strengthened through more supportive supervision and regular meetings with VHT colleagues to address work challenges; additional support and training to improve VHTs’ awareness and navigation of ethical dilemmas should be incorporated; and enhanced integration of VHTs into the health system, including more respectful treatment by other health professionals should take place. This study examines how VHTs negotiate their roles as insiders, outsiders, and intermediaries, and the way these fluid positions confer both access and benefits, as well as barriers and challenges to their work.

## Additional file

Additional file 1: VHT Interview Guide. File includes the interview guide developed for this project. (DOCX 25 kb)

## Data Availability

The interview guide used and transcripts analysed during the current study are available from the corresponding author on reasonable request.

## Abbreviations

ACCESS: African Community Center for Social Sustainability
CHW: Community Health Worker
SRH: Sexual and reproductive health
VHT: Village Health Team
