# Supplementary material for: Outsiders, insiders, and intermediaries: village health teams’ negotiation of roles to provide high quality sexual, reproductive and HIV care in Nakaseke, Uganda
Source: BMC Health Serv Res. 2019 Aug 13;19:563. doi: 10.1186/s12913-019-4395-4 (PMC6692941; doi:10.1186/s12913-019-4395-4)
Supplement: Supplementary file 1 — VHT Interview Guide. File includes the interview guide developed for this project. (DOCX 25 kb) [file 12913_2019_4395_MOESM1_ESM.docx]

**VHT Interview Guide**

Respondent Code:

Interviewer Code:

[Guidance for interviewer: PLEASE START RECORDING IMMEDIATELY **AFTER** you have received the consent of the interviewee. Please read the following statement.]

We would like to learn about your experiences as a VHT member. Thank you for agreeing to help us learn about this topic.

[Guidance for interviewer: For this first section, please write down the answers that the interviewees give to these short questions.]

**PART 1: SOCIO-Demographic Background**

| **[Research Question (RQ) 2] Questions** | **Answers** | **Problem?** |
| --- | --- | --- |
| 1. How old are you? | _______ years old |  |
| 1. [Interviewer, please note gender] | 0= Female  1= Male |  |
| 1. What is your marital status? | 1=Married  2=Unmarried  3=Widowed  4=Divorced |  |
| 1. Do you have any children? | 0= No  1= Yes |  |
| 1. What was your level of education | 1=None  2=Some primary  3=Completed primary  4=Some secondary  5=Completed Secondary  6=Post-Secondary |  |
| 1. What is your religion?   Please circle only one answer: | 1 = Roman Catholic  2 = Anglican  3 = Pentecostal  4 = Muslim  5 = Traditional  5 = Other, specify __________________  6 = None |  |

**Introduction/Clinical Experience**

How long have you been a VHT member?

Why did you decide to become a VHT member?

**Preparation/Knowledge**

What training have you received regarding:

- How to talk to clients about health in general
- How to talk to clients about sensitive topics such as sexual health
- Work with clients who are HIV positive or at risk for HIV
- Patients’ rights or professional ethics

[RQ 1] What rights do you believe patients have when they are receiving health care?

[Guidance for the interviewer: Please check any mention of the following terms]

- Patient privacy and confidentiality ensured at all times
- Respect shown to client/patient as an individual
- Information given to client is accurate and unbiased
- Client/patient allowed to make her own decisions and choose her own care

**Procedure/Perceived Role**

In a typical day, what do you do as a member of the VHT?

- Probe: what are your responsibilities as a VHT?

[RQ 3] What is your role in working with community members living with HIV?

- Probe for women

[RQ 3 ] What do you see as your role in promoting reproductive health? (Probe for how they explain their role to clients)

[RQ 3] How would you handle an individual who doesn’t know her HIV status and doesn’t want to know?

[RQ 3] How would you handle a woman/man who is HIV+ and doesn't wish to tell her husband/partner or family?

- What would you do if a client with HIV does not follow up with their appointments and medication?
- What poses the greatest challenge to your work in trying to prevent HIV?
  - *probing for individual and contextual factors

**Experience with Tension/Recommendations**

[RQ 3] What’s it like for you as a community member to also be a member of the VHT?

- Do you feel any tensions there? Please elaborate- give an example, etc.

[RQ 3] Have you ever had objections from the community during your work as a VHT?

- If yes, please explain?

[RQ 3] What does your family think of your role as a VHT worker?

[RQ 3] What do you like best about being a VHT? (Probe for feeling appreciated)

What is the one thing that you think would most improve your work as a VHT?

Is there anything else you’d like to share about being at VHT?

Thank you for your time.
